# A QTL associated with leaf trichome traits has a major influence on the abundance of the predatory mite *Typhlodromus pyri* in a hybrid grapevine population

**DOI:** 10.1038/s41438-019-0169-8

**Published:** 2019-07-21

**Authors:** Paola Barba, Rebecca Loughner, Karen Wentworth, Jan Peter Nyrop, Gregory M. Loeb, Bruce I. Reisch

**Affiliations:** 1 Plant Breeding and Genetics Section, School of Integrative Plant Science, Cornell University, Ithaca, NY 14853 USA; 2 Department of Entomology, Cornell AgriTech, Geneva, NY 14456 USA; 3 Horticulture Section, School of Integrative Plant Science, Cornell AgriTech, Geneva, NY 14456 USA; 4 Present Address: Instituto de Investigaciones Agropecuarias, INIA La Platina, Santiago, 8831314 Chile

**Keywords:** Plant breeding, Genetic markers, Plant breeding, Agricultural genetics

## Abstract

The abundance of predatory phytoseiid mites, *Typhlodromus pyri*, important biological control agents of spider mite pests in numerous crops, is positively influenced by the density of leaf trichomes and tuft-form domatia in vein axils. Identification of the genetic regions controlling both trophic levels could facilitate the improvement of predatory mite habitat in breeding programs. The abundance of *T. pyri* and non-glandular trichomes was measured in a segregating F_1_ family derived from the cross of the complex *Vitis* hybrid, ‘Horizon’, with Illinois 547-1 (*V. rupestris* B38 × *V. cinerea* B9), finding positive correlation among traits. High density genetic maps were used to localize one major quantitative trait locus (QTL) on chromosome 1 of Illinois 547-1 associated with both predatory mite abundance and leaf trichomes. This QTL explained 23% of the variation in phytoseiid abundance and similar amounts of variance in domatia rating (21%), domatia size (16%), leaf bristle density (37% in veins and 33% in blades), and leaf hair density (20% in veins and 15% in blades). Another nine QTL distributed among chromosomes 1, 2, 5, 8, and 15 were associated solely with trichome density, and explained 7–17% of the phenotypic variation. Combined, our results provide evidence of the genetic architecture of non-glandular trichomes in *Vitis*, with a major locus influencing trichome densities, domatia size and predatory mite abundance. This information is relevant for breeding grapevines with a more favorable habitat for biological control agents.

## Introduction

Leaf morphological traits in plants can positively or negatively affect plant fitness through a direct effect on the second trophic level (plant feeding insects) and indirectly by affecting the abundance of the third trophic level (natural enemies of plant feeding insects) (tri-trophic interactions sensu^1^). Leaf trichomes have been particularly well studied in this regard. In multiple plant systems, leaf trichomes have a direct negative effect on the performance of small herbivorous arthropods (direct defense)^2–7^. This includes both non-glandular epidermal trichomes (bristles and hairs) and glandular trichomes that release sticky and/or toxic substances^8^. In some cases, leaf trichomes positively affect preference or performance of herbivorous arthropods, although more commonly, performance can be enhanced indirectly by negatively affecting performance and predation rates of natural enemies^9^. This is best shown with glandular trichomes^8,10–12^. The relationship between non-glandular trichomes and natural enemies such as predatory and mycophagous mites has frequently been shown to be positive (reviewed in refs. ^13,14^) although counter examples also exist^14–17^.

The mechanistic basis of the positive direct effect of non-glandular trichomes on natural enemy mites is most likely related to providing shelter from their natural enemies (intraguild predators), but also trichomes may increase the availability of alternative food sources such as pollen (reviewed in ref. ^14^). Non-glandular trichomes can be widely distributed on the leaf blade, or concentrated along veins or in vein axils on the abaxial leaf surface. Non-glandular trichomes concentrated in the vein axils are referred to as tuft-form acarodomatia to reflect the common observation that they are frequently occupied by predatory and mycophagous species of mites as opposed to phytophagous species^13,18–20^. Trichomes that make up tuft-form acarodomatia (simplified from here on as domatia) appear similar in shape and size to non-glandular trichomes found on leaf veins or the leaf blade, although the underlying embryogenesis or genetics have not been explicitly explored.

Predatory mites in the families Phytoseiidae and Stigmaeidae are important biological control agents of spider mites and other arthropod pests in agriculture^21,22^, and mycophagous mites in the family Tydeidae are important consumers of some fungal pathogens such as grape powdery mildew^23^. *Typhlodromus pyri* are generalist phytoseiids that prey on grapevine pests, including the European red mite *Panonychus ulmi* and the grape rust mite *Calepitrimerus vitis* (Nalepa)^22,24^, but also feed on pollen and fungal spores. The presence of trichomes and domatia can influence the abundance of some species of generalist phyotoseiid mites even greater than the abundance of prey^25–27^. Generalist phytoseiids often make greater use of and lay more eggs on plants with pubescent leaves, compared with plants with glabrous leaves^28^. *T. pyri* and some other generalist phytoseiid mites show a strong preference for leaves containing non-glandular trichomes and are more abundant in grape and apple cultivars with pubescent leaves^29,30^. In an assemblage study, plant patches containing higher proportions of glabrous plants had significantly lower populations of *T. pyri* 6 weeks after inoculation compared with assemblages with higher proportions of pubescent plants, suggesting that trichomes are required to maintain the overall abundance of these predatory mites^31^.

Genes regulating the development of non-glandular trichomes in the model plant *Arabidopsis thaliana* are well characterized^32,33^. The GLABROUS2 (GL2) homeodomain protein, required for trichome morphogenesis, can be activated or repressed by several transcription factors (Table 1), which result in either positive or negative effects, respectively, on trichome development.

**Table 1:** List of proteins regulating GLABROUS2 (GL2)

Function	Protein type	Protein name
Part of GL2 activator complex	WD40	TRANSPARENT TESTA GLABRA 1 (TTG1)
	R2R3 MYB	GLABROUS1 (GL1), MYB23 and MYB5
	bHLH	GLABROUS3 (GL3), ENHANCER OF GLABROUS3 (EGL3), TRANSPARENT TESTA (TT8), and MYC-1
Negative regulation of GL2	R3 MYB	TRIPTYCHON (TRY), CAPRICE (CPC), ENHANCER OF TRY and CPC1 (ETC1, ETC2, ETC3), and TRICHOMELESS1 (TCL1, TCL2)
Gibberellic acid-dependent negative regulators	DELLA	GA INSENSITIVE (GAI)
		REPRESSOR OF gal-3 (RGA)
		RGL1, RGL2, and RGL3
	C2H2 zinc finger proteins (ZFP)	GLABROUS INFLORESCENCE STEMS (GIS, GIS2), ZFP8, ZFP5, and ZFP6

The protein GL2 is required for trichome morphogenesis in *Arabidosis thaliana*, the proteins listed below can have either a positive or negative effect on trichome development through GL2 regulation

The heritability of domatia size^26^ and trichome density^34–36^ have been demonstrated in *Vitis* and several other species. But despite the evidence of genetic determinism of trichomes and domatia, and the positive correlation with predatory mite abundance, these phenotypes have not been widely employed in plant breeding programs though they may be used effectively to improve predatory mite habitats^14^.

In order to achieve enhanced biological control through breeding, there are two main aspects that require further attention. First, we need to determine and understand how the desirable trait (i.e., increasing the size of predatory mite populations) is inherited. Second, we need to determine if the progress achieved through selection is sufficient to provide effective biological pest control. In this paper we address the first question by studying the genetics of predatory mite abundance and leaf trichomes in a cross of two grapevine hybrids ‘Horizon’ and Illinois 547-1. We first studied the segregation and correlation between phytoseiid abundance and leaf morphological traits (domatia, bristles and hair on leaf veins and blades). Then, we localized and characterized the regions of the genome controlling these traits by multiple QTL mapping.

## Results

To test whether predatory mite abundance and density of leaf morphology traits such as domatia, bristles and hairs have common genetic factors, we characterized parental vines and their F_1_ family segregating for these traits.

### Characterization of parental leaf trichome traits

**Fig. 1:**
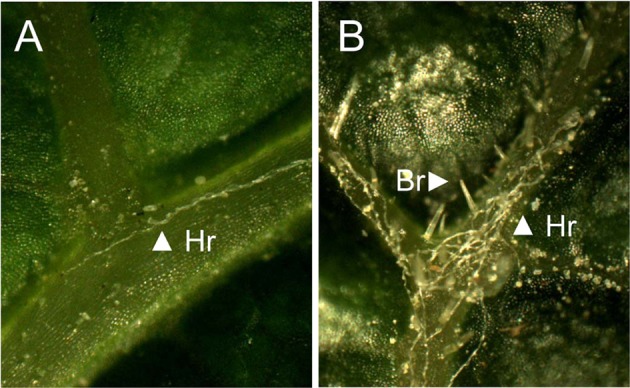
Comparison of densities of bristles and hairs on leaf veins. For parental genotypes ‘Horizon’ (**a**) and Illinois 547-1 (**b**). Examples of bristles (Br) and hairs (Hr) are indicated

Parental genotypes ‘Horizon’ (glabrous) and Illinois 547-1 (pubescent) expressed different levels of leaf trichomes (Fig. 1). Observations showed clear variation in the density of bristles and hairs in both leaf blade and leaf veins (Table 2).

**Table 2:** Leaf morphological traits for parental grapevines ‘Horizon’ and Illinois 547-1

Trait	‘Horizon’	Illinois 547-1	*p*-value
Domatia size (mm)	0.95	1.27	0.11
Domatia (rating)	7.5	8.5	<0.0001
Bristles on leaf veins (density)	1.8	7.9	<0.0001
Bristles on leaf blade (density)	0.2	4.0	<0.0001
Hairs on leaf veins (density)	4.0	3.1	0.013
Hairs on leaf blade (density)	3.6	0.0	<0.0001

Values correspond to the means of ten independent leaves. The density of bristles and hairs was measured on a discrete scale from 0 (absent) to 9 (very dense), and four domatia per leaf were measured and rated with the bristle scale. Significance of differences were obtained by a *t*-test

### Characterization of leaf trichome traits and predatory mite abundance in an F_1_ family

**Fig. 2:**
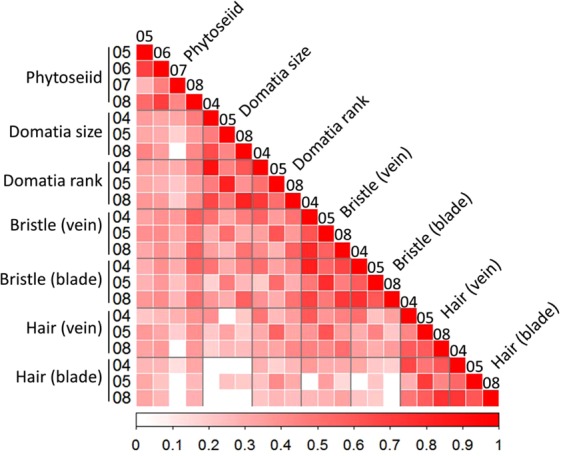
Correlation of phytoseiid abundance and leaf traits in F_1_ progeny of ‘Horizon’ × Illinois 547-1

Phytoseiid abundance on F_1_ individuals was positively correlated among the 4 years, and also positively correlated with all leaf morphology traits (Fig. 2). The correlation of phytoseiid abundance measured in 2007 was weaker, but still significant and positive with at least 1 year of evaluation of all other traits.

Pearson’s correlations among phenotype by year are represented by a color scale. Non-significant correlations (at *α* = 0.05) are indicated in white. No negative correlations were found.

Leaf morphology traits were positively correlated among each other in all cases, showing stronger correlations with data of a similar nature, such as domatia size with domatia rating, bristle density on veins with bristle density on blades, and hair density on veins with hair density on blades. There was also a strong correlation among phenotypes measured in the same year. Hair phenotypes showed the weakest correlations with other morphology traits (always positive but some non-significant), particularly with domatia size and bristle density on leaf blades.

**Fig. 3:**
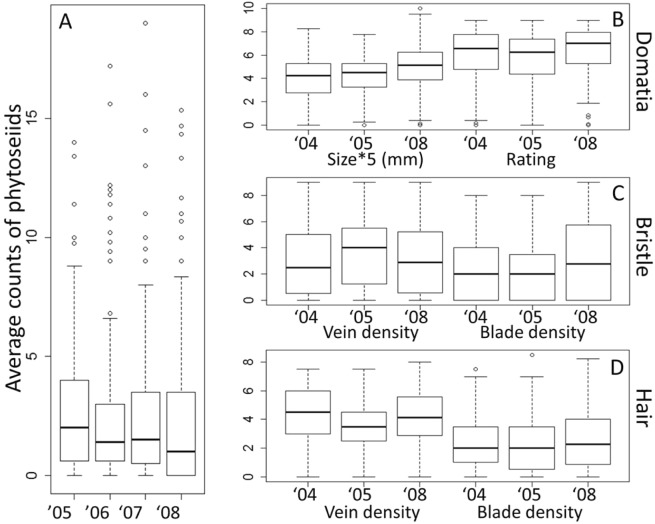
Phenotype distributions (box and whiskers plots). **a** Predatory mite abundance (mean phytoseiid count per leaf), **b** domatia size and rating, **c** bristle density in veins and blades, and **d** hair density in veins and blades, by year. Domatia size is shown as 5X mm measures to fit the plot scale. Bristle and hair density for both veins and blades were determined using a rating system from 0 (absent) to 9 (very dense). Domatia ratings correspond to the average bristle density rating of four domatia per leaf. For each data set, the box indicates the range between 25 and 75%, and middle band indicates the sample median. Outlier values are indicated with a circle

Leaf morphological traits showed continuous and consistent segregation patterns across years, with individuals representing the spectrum of ratings from 0 (absent) to near 9 (very dense) (Fig. 3). Both domatia measures showed the shortest interquartile range, consistent with similar mean values obtained on both parents. Bristle densities were similar on both leaf veins and leaf blades, with a higher proportion of individuals with zero bristles on blades. Hair density distribution was different on leaf veins than on leaf blades.

### QTL detection

To find the genetic regions controlling the phenotypes described above, and to assess to what extent distribution of phytoseiid abundance and trichome densities in the ‘Horizon’ × Illinois 547-1 family is due to major genetic components, we conducted a multiple QTL mapping study.

QTL models were significant for each trait in each year where data were collected, explaining between 11.1 and 69.4% of the phenotypic variance. Among all traits, a total of 16 QTL were significant for 2 or 3 years and were distributed among chromosomes 1, 2, 5, 8, and 15, with chromosomes 1, 5, and 15 contributing effects from both parental maps (Table 3). For all traits, 14 QTL were above threshold for only one evaluation date. These unstable QTL are indicated in Table 3, but were not considered for further analysis.

**Table 3:** QTL models for phytoseiid counts, domatia size and rating, bristle, and hair densities

Phenotype	Year	Sample size	PVE^a^	Model
Phytoseiid (counts)	2005	147	23.8	20@37.9 + 37@0.9^b^
	2006	147	24.7	20@40.6
	2007	143	11.1	20@32.4
	2008	152	54.0	20@37.3 + 1@68.3^b^
Domatia (rating)	2004	190	38.5	20@42.0 + 34@29.0
	2005	155	14.5	20@37.6
	2008	152	46.8	20@41.0 + 34@29.8 + 18@32.0^b^ + 1@47.0^b^
Domatia (mm)	2004	190	43.0	20@37.0 + 34@31.0 + 23@12.7^b^
	2005	154	13.0	20@37.6
	2008	152	50.9	20@38.3 + 34@22.0 + 10@9.7^b^ + 18@20.7^b^ + 37@24.6^b^
Bristles on veins (density)	2004	190	63.2	20@41.8 + 1@67.2 + 8@72.4^b^
	2005	155	44.1	20@37.6 + 1@56.0
	2008	152	61.8	20@47.3 + 1@65.8
Bristles on leaf blades (density)	2004	190	55.0	20@41.8 + 1@77.5 + 15@27.9
	2005	155	42.7	20@43.0 + 19@69.9^b^ + 1@61.0
	2008	152	69.4	20@45.0 + 15@26.0 + 1@68.3 + 36@41.0^b^
Hair on leaf veins (density)	2004	190	25.8	20@43.5
	2005	155	31.9	20@37.6 + 35@62.0^b^ + 2@50.0
	2008	152	40.2	20@35.0 + 27@56.7^b^ + 2@47.0
Hair on leaf blades (density)	2004	190	49.5	24@5.0 + 20@42.2 + 5@4.3 + 35@65.0^b^ + 27@60.3 + 24@5.0 : 5@4.3^b^
	2005	155	15.6	24@5.0
	2008	152	41.7	20@34.4 + 27@56.7 + 5@3.1 + 24@4.0

QTL were named using R/qtl nomenclature, i.e., linkage group@position (cM). Linkage group numbers 1–19 correspond to chromosome numbers in the female map (‘Horizon’), and 20–38 correspond to chromosome numbers 1–19 in the male map (Illinois 547-1)

: Indicates an interaction term between two QTL

^a^PVE, percentage of variance explained by the model

^b^QTL that were significant only on one date of evaluation

### Co-localization of mite abundance and trichome traits

Of all QTL, one on chromosome 1 from Illinois 547-1 explained a higher percentage of the phenotypic variance, and was the only QTL also associated with predatory mite abundance. This QTL was significant for 4 consecutive years (2005–2008) and it co-localized with major QTL for each leaf morphology trait (Table 4).

**Table 4:** Statistics of stable QTL

Chr^a^	Parent	Phenotype	Year	Position of LOD peak and supported interval (cM)	LOD	LOD Th^b^	PVE^c^	Effect	Associated marker and alleles^d^
1	Illinois 547-1	Phytoseiid (counts)	2005	36.2–37.9–45.0	5.36	3.42	13.9	2.41	S1_10602967 A/G
			2006	35.3–40.6–43.5	9.06	3.38	24.7	3.25	
			2007	29.1–32.4–58.0	3.64	3.46	11.1	2.32	
			2008	36.2–37.3–41.8	21.0	3.49	41	3.77	
1	Illinois 547-1	Domatia (rating)	2004	35.3–42.0–45.3	15.5	3.34	28	2.24	S1_11789500 A/T
			2005	35.3–37.6–49.9	5.25	3.33	14.5	1.62	
			2008	35.3–41.0–46.0	10.3	3.25	19.6	1.67	
1	Illinois 547-1	Domatia (mm)	2004	35.3–37.0–49.0	11.9	3.45	19	0.28	S1_10130853 G/A
			2005	35.3–37.6–48.5	4.65	3.35	13	0.25	
			2008	36.2–38.3–42.6	8.67	3.42	14.7	0.33	
1	Illinois 547-1	Bristle on veins (density)	2004	41.0–41.8–45.0	31.5	3.46	42.1	3.81	S100_486628 C/–
			2005	36.2–37.6–43.5	15.4	3.3	32.3	3.17	
			2008	44.0–47.3–47.9	22.5	3.42	37.3	3.61	
1	Illinois 547-1	Bristle on leaf blades (density)	2004	40.6–41.8–45.0	26.1	3.36	39.7	2.91	S100_311430 A/G
			2005	36.2–43.0–51.0	11.0	3.39	21.6	2.26	
			2008	43.5–45.0–46.7	26.9	3.43	38.4	3.79	
1	Illinois 547-1	Hair on leaf blades (density)	2004	36.2–42.2–45.0	9.31	3.44	12.8	1.29	S1_12478623 C/A
			2005	36.2^e^	2.31^e^	3.32	n.s.	n.s	
			2008	29.8–34.4–47.3	8.32	3.29	16.7	1.71	
1	Illinois 547-1	Hair on leaf veins (density)	2004	40.6–43.5–45.0	12.3	3.29	25.8	1.70	S1_10130853 G/A
			2005	33.0–37.6–48.5	6.11	3.33	13.6	1.32	
			2008	32.0–35.0–45.0	9.83	3.32	20.8	1.59	
1	'Horizon'	Bristle on veins (density)	2004	58.0–67.2–83.1	6.47	3.46	6.25	1.96	S1_18418322 A/T
			2005	47.0–56.0–83.1	4.39	3.3	7.78	1.74	
			2008	60.0–65.8–83.0	11.7	3.42	16.2	2.64	
1	'Horizon'	Bristle on leaf blades (density)	2004	62.0–77.5–83.1	5.12	3.36	5.95	1.41	S1_18418322 A/T
			2005	50.0–61.0–83.1	4.31	3.39	6.74	1.51	
			2008	59.0–68.3–82.0	8.21	3.43	8.63	2.52	
2	'Horizon'	Hair on leaf veins (density)	2004	57.4^e^	.20^e^	3.29	n.s.	n.s	S2_13348927 T/G
			2005	37.4–50.0–62.4	3.46	3.33	7.37	0.94	
			2008	35.0–47.0–57.4	3.79	3.32	7.29	1.08	
5	'Horizon'	Hair on leaf blades (density)	2004	0.00–4.29–7.89	7.7	3.44	6.91	0.95	S5_2523862 A/T
			2005	3.13^e^	1.28^e^	3.32	n.s.	n.s	
			2008	0.00–3.13 – 12.5	4.46	3.29	8.43	1.34	
5	Illinois 547-1	Hair on leaf blades (density)	2004	1.13 – 5.00 – 6.00	11.5	3.44	16.2	1.32	S5_750796 T/C
			2005	0.74–5.00–10.0	5.69	3.32	15.6	1.61	
			2008	0.00–4.00–8.00	4.42	3.29	8.35	1.41	
8	Illinois 547-1	Hair on leaf blades (density)	2004	53.0–60.3–63.7	5.29	3.44	7.36	0.84	S8_19609227 –/C
			2005	54.9^e^	1.30^e^	3.32	n.s.	n.s	
			2008	47.3–56.7–62.0	4.57	3.29	8.65	1.33	
15	'Horizon'	Bristle on leaf blades (density)	2004	24.0–27.9–39.4	4.13	3.36	4.74	0.94	S15_14485614 C/T
			2005	27.9^e^	1.68^e^	3.39	n.s.	n.s	
			2008	24.0–26.0–30.0	9.64	3.43	10.4	1.97	
15	Illinois 547-1	Domatia (rating)	2004	26.0–29.0–43.0	8.60	3.34	14.4	1.51	S15_14939741 A/G
			2005	29.8^e^	1.00^e^	3.33	n.s.	n.s	
			2008	23.0–29.8–32.0	10.3	3.25	19.5	1.40	
15	Illinois 547-1	Domatia (mm)	2004	25.1–31.0–34.9	11.5	3.45	18.4	0.28	S15_15178923 A/G
			2005	28.6^e^	0.53^e^	3.35	n.s.	n.s	
			2008	18.5–22.0–28.0	6.1	3.42	9.97	0.28	

QTL detected for 2 or 3 years following 4 years of data collection for phytoseiid counts and 3 years of data collection for trichome-related traits. QTL were obtained by multiple QTL mapping on separated parental maps. QTL effects are measured in the phenotype scale indicated, where densities and ratings correspond to the scale from (0) to (9). Alleles of an associated marker are indicated according to the QTL effect, with the allele with higher mean phenotype value first

n.s.: Values not reported as QTL were not significant for this year

^a^Chr corresponds to the physical chromosome number

^b^LOD threshold, was determined by permutation test (1000) over the whole set of markers, at *α* = 0.05

^c^PVE, percentage of the total phenotypic variance explained by a single QTL

^d^Marker name indicates the position on the 12X.0 PN40024 *Vitis vinifera* reference genome in format S(chromosome)_(position in bp)

^e^No significant hit for this year. The highest LOD score by single QTL scan is reported

Statistics associated with this QTL were consistent among years, with some variation in the QTL peak, supported interval and effect. According to the physical coordinates of flanking SNPs on chromosome 1, the phytoseiid QTL peak was located between 8,744,147 and 11,789,500 bp. For domatia size and rating, QTL peak ranges were similar, between 9,877,466 and 10,879,085 bp and between 9,982,106 and 14,859,772 bp, respectively. For other trichome traits, the range of the QTL peaks was wider, but still within the supported interval of the QTL associated with predatory mite abundance. Density of bristles on leaf veins and leaf blades showed QTL peaks between 10,130,832 and 18,359,738 bp, and between 12,043,160 and 15,492,583 bp, respectively. Density of hairs on leaf veins and leaf blades were associated with QTL peaks between 8,744,147 and 14,859,772 bp, and between 9,395,874 and 15,492,583 bp, respectively.

### Additional QTL for domatia and trichome phenotypes

In addition to the major QTL on chromosome 1, other QTL were associated solely with leaf trichomes. A QTL located on chromosome 15 was significant for domatia rating and domatia size. For bristle density, a minor QTL located on chromosome 1 of ‘Horizon’ was associated with variation in both leaf veins and leaf blades. Another minor QTL, only for bristles density on leaf blades, was located on chromosome 15 of ‘Horizon’. Different sets of minor QTL were found for hair density on veins versus leaf blades (Table 4).

### Candidate genes

Reference genome-based genotyping-by-sequencing directly produces physical positions for QTL support intervals on a genetic map, facilitating the search for candidate genes.

We looked for evidence of *Vitis* candidate genes with similarity to 28 *Arabidopsis thaliana* genes involved in the development of non-glandular trichomes (Additional file 1), using the PN40024 reference genome regions delimited by all QTL intervals. Twelve such candidate genes were identified, including three located on chromosome 1 and five on chromosome 15 (Table 5 and Additional file 2).

**Table 5:** Grapevine candidate genes for phytoseiid abundance and trichome related traits

Chr^a^	Start position	*Vitis* gene name^b^	Trichome-related gene name; function; Query id	Associated phenotype
1	8,079,551	VIT_201s0127g00730	GL1 (GLABRA 1); transcription factor; NP_189430.1	Phytoseiid, hair on blades
			MYB23 (myb domain protein 23); DNA binding/transcription factor; NP_198849.1	
	9,724,089	VIT_201s0026g00850	GIS2 (GLABROUS INFLORESCENCE STEMS 2); nucleic acid binding/transcription factor/zinc ion binding; NP_196283.1	Phytoseiid, domatia (rating), domatia (size), hair on blades, hair on veins
			ZFP8 (ZINC FINGER PROTEIN 8); nucleic acid binding/transcription factor/zinc ion binding; NP_181725.1	
	19,230,746	VIT_201s0010g02270	RGA1 (REPRESSOR OF GA1-3 1); transcription factor; NP_178266.1	Phytoseiid, domatia (rating), bristles on blades, bristles on veins
			RGL1 (RGA-LIKE 1); transcription factor; NP_176809.1	
2	8,739,817	VIT_02s0012g02030	GL2 (GLABRA 2); DNA binding/transcription factor; NP_001185443.1	Hair on veins
5	1,128,866	VIT_05s0077g01390	GIS2 (GLABROUS INFLORESCENCE STEMS 2); nucleic acid binding/transcription factor/zinc ion binding; NP_196283.1	Hair on blades
8	20,543,918	VIT_208s0007g06870	GIS (GLABROUS INFLORESCENCE STEMS); nucleic acid binding/transcription factor/zinc ion binding; NP_191366.1	Hair on blades
			GIS2 (GLABROUS INFLORESCENCE STEMS 2); nucleic acid binding/transcription factor/zinc ion binding; NP_196283.1	Hair on blades
			ZFP8 (ZINC FINGER PROTEIN 8); nucleic acid binding/transcription factor/zinc ion binding; NP_181725.1	Hair on blades
	20,865,263	VIT_208s0007g07230	MYB5; transcription repressor; NP_187963.1	Hair on blades
15	13,245,041	VIT_15s0021g02290	SPL8 (SQUAMOSA PROMOTER BINDING PROTEIN-LIKE 8); DNA binding; NP_683267.1	Domatia (size)
	13,256,743	VIT_215s0021g02300	SPL8 (SQUAMOSA PROMOTER BINDING PROTEIN-LIKE 8); DNA binding; NP_683267.1	Domatia (size)
	16,133,315	VIT_15s0048g02000	GL2 (GLABRA 2); DNA binding/transcription factor; NP_001185443.1	Domatia (size), domatia (rating), bristles on blades
	17,211,622	VIT_215s0046g00170	MYB5, transcription repressor; NP_187963.1	Domatia (rating)
	18,177,217	VIT_215s0046g01130	CPC (CAPRICE); DNA binding/transcription factor; AAS09991.1	Domatia (rating)
			ETC2 (ENHANCER OF TRY AND CPC 2); DNA binding/transcription factor; NP_850145.1	Domatia (rating)
			ETC3 (ENHANCER OF TRY AND CPC 3); DNA binding/transcription factor; NP_974493.1	Domatia (rating)
			TCL1 (TRICHOMELESS1); DNA binding; NP_001031445.1	Domatia (rating)
			TCL2 (TRICHOMELESS2); NP_001118417.1	Domatia (rating)
			TRY (TRIPTYCHON); DNA binding transcription factor; NP_200132.2	Domatia (rating)

Candidate genes based on the PN40024 *Vitis vinifera* 12X.0 reference genome. Homologous *Arabidopsis thaliana* genes are indicated

^a^Chr indicates the physical chromosome number

^b^*Vitis* gene names come from annotations V1 and V2.1 from the CRIBI database^37^

## Discussion

The abundance of the predatory mite, *T. pyri*, on progeny of ‘Horizon’ and Illinois 547-1 grapevines was significantly associated with a region of the Illinois 547-1 genome that also had a major effect upon densities of domatia, hairs, and bristles. While a molecular mechanism for trichome development is well-established in model plants, as is the positive relationship of leaf trichome density and predatory mite abundance, the location of a QTL positively associated with predator abundance in a different trophic level is uncommon. Our results shed light on the genetic control of leaf landscape features for predatory mite habitat enhancement.

Our phenotypic assessment showed that predatory mite abundance was positively correlated with all trichome traits. Among those, bristles and domatia showed the highest correlation, and to a minor degree with leaf hair. These phenotypic results correspond with the genetic loci found on chromosome 1 of Illinois 547-1, explaining a major proportion of the variance observed in phytoseiid abundance, domatia and bristle traits, and to a lesser degree, in hair densities. Previous ecological studies^30^ reported a similar correlation of phytoseiids with trichomes in a sample of 12 distinct grapevine cultivars. There, phytoseiid abundance was best predicted by a model in which domatia and hair density had an additive effect and domatia had the greatest explanatory power followed by hair density.

The genetic architecture of trichome traits, with a major QTL also associated with phytoseiid abundance, and different sets of minor QTL for trichomes on leaf blade or veins, may explain the correlation observed between mite abundance and leaf trichomes in previous analyses^14^. This may suggest that either a single gene, or several genes from either the trichome developmental pathway or for general leaf morphology, may be linked on chromosome 1. Whether these two pathways are connected cannot be elucidated with this experimental design.

The association between general leaf morphology traits and leaf hair was described in a meta-analysis of at least 117 *Vitis* accessions^36^. The authors suggested that aspects of vein patterning, laminar outgrowth and epidermal features such as hair, may be regulated by common developmental pathways. In their work, a QTL for the first principal component of leaf morphology was found on chromosome 1, about 4.2 Mb and 16.6 cM (as measured on the Illinois 547-1 genetic map) from the major QTL for predatory mite and trichome traits found in the present study.

Moreover, two predicted *Vitis* genes located within the major QTL region and near its LOD peak, showed homology to components of the trichome regulatory pathway. VIT_201s0127g00730 was similar to transcription factors of the GL2 activator complex (GL1/MYB23) and VIT_201s0026g00850 to gibberellic acid-dependent upstream repressors of the GL2 activator complex (GIS2/ZFP8). These are interesting candidate genes within a large genetic region, as the F_1_ family used in this study has limited resolution to accurately propose a causal gene. To elucidate the genetic control of the predatory mite abundance QTL, more recombination around this locus is needed. A larger F_1_, F_2_, or an association mapping population would be more suitable to narrow down the position of the locus and further reduce the list of candidate genes.

Among the progeny, phytoseiid counts and trichome density segregated continuously. This may indicate a quantitative genetic architecture of the trait and an environmental effect on its expression. QTL models were able to explain only a portion of all phenotypic variance. The percent of variance explained varied among years, suggesting either an environmental effect in the expression of these traits or sampling errors. This is also evident from the appearance of unstable QTL, which were significant only in 1 year. Despite year-by-year variation, the trait distribution means were fairly stable, and 16 QTL were detected in more than one season, with overlapping QTL supported intervals.

Overall, bristles on leaf veins had the simplest genetic model. On average, 56.5% of the variance was explained by the additive effects of two QTL on chromosome 1 of each parental map. In addition, bristles on leaf blades were controlled by a third locus located on chromosome 15 of ‘Horizon’. An additive model with these three loci explained a similar proportion of the phenotypic variance.

Domatia are leaf structures primarily comprised of bristles. QTL for domatia were co-localized with QTL for bristles on leaf blades, segregating on the Illinois 547-1 map. Together, these two QTL explained a lower proportion of the total variance for domatia, 35.6 and 33.3% when measured by size or by a rating, respectively. Measuring domatia size is a direct quantification of the phenotype, but it is labor intensive and may be more prone to human error. In this research, both scales led to similar QTL results (numbers of QTL and total variance explained). Our results suggest that it is reasonable to apply a categorical scale for QTL mapping of this difficult-to-quantify trait, until more precise quantitative techniques are developed.

The genetic architecture of hair trichomes was more complex, showing greater differences in the number and position of loci controlling the density of hairs on either leaf veins or leaf blades. Such findings are consistent with the dissimilarities in the phenotype segregation. Hairs on leaf veins showed a simpler genetic model, including the major QTL on chromosome 1 and a second QTL on chromosome 2. Together, these QTL explained an average of 32.7% of the variance. In contrast, hair located on leaf blades was associated with four QTL: the major QTL on chromosome 1 of Illinois 547-1; a minor QTL on chromosome 8 of the Illinois 547-1 map; and two co-localized QTL on chromosome 5 (one on each parental map). Within the latter two QTL regions, candidate genes VIT_05s0077g01390 and VIT_208s0007g06870, with homology to proteins implicated in cell differentiation (GIS and GIS2) were found. These QTL on chromosome 5 and 8 were also detected using image-based phenotyping in a previous report^38^.

Being on a different trophic level, predatory mite abundance is more prone to environmental influence. This phenotype was more variable and presented more extreme observations. The amount of phenotypic variance explained by genetics was lower than for domatia and trichome traits, with values ranging from 11.1% in 2007 to 54.0% in 2008. The most stable locus co-localized with the major QTL for domatia and all trichome traits on chromosome 1 of Illinois 547-1, but in 2008 a QTL on chromosome 1 of the ‘Horizon’ parent was also significant. The position of the Illinois 547-1 QTL was estimated around 37 cM (10 Mbp in the 12x.0 version of the PN40024 reference genome) on chromosome 1, with an average effect of three mites per leaf, and mean values ranging from 2.3 in 2007 to 3.8 in 2008. These results indicate that, despite the year-to-year fluctuations of phytoseiid counts, it is still possible to explain a difference of three mites per leaf with only one polymorphism at the locus.

Breeding for domatia and trichome densities could be an indirect form of breeding for resistance to pests, by promoting the abundance of generalist feeding phytoseiids in new cultivars. Here, we demonstrated that a single locus in the host genome can produce significant differences in the abundance of predatory mites. Selection for this QTL will likely increase the overall density of domatia, bristles and hair, which could have a pleiotropic effect on other plant functions such as on pests not controlled by *T. pyri* or other natural enemies of insect and mite pests^9^.

The identification of stable genetic regions along with their positions on a reference genome may help to accelerate further discoveries, by providing linked molecular markers and regions to search for candidate genes. Molecular markers identified in the present project may allow breeders to identify progeny shortly after seed germination that will provide better habitats for predatory mites. These can be used in combination with markers for other relevant traits, such as flower sex^39^, disease resistance^40,41^ or improved berry and cluster architecture^42,43^. Moreover, the additive effects of the QTL suggest that stacking alleles and loci with minor effects could increase the numbers of phytoseiids per leaf, in order to sustain higher population of predatory mites and therefore more consistent control of pest mites.

Correlation between predatory mite (*T. pyri*) abundance and the presence of non-glandular trichomes on grapevine leaves can be explained by the co-segregation on chromosome 1 of quantitative trait loci associated with these traits. The genetic architecture of leaf domatia, bristles and hairs is characterized by the additive effect of a major QTL, plus other minor QTL explaining positional effects. By demonstrating that the abundance of a biological control agent can be influenced by the genetics of its host through modification of its leaf landscape, our results provide information for breeding grapevines with a more favorable habitat for important biological control agents.

## Materials and methods

### Plant material

A set of 190 seedlings was generated from crosses made in 1988 and 1996 between the complex grapevine hybrid ‘Horizon’ (‘Seyval’ × ‘Schuyler’, whose pedigree includes *Vitis vinifera*, *V. labrusca, V. aestivalis*, and *V*. *rupestris*) and Illinois 547-1 (*V*. *rupestris* B38 × *V*. *cinerea* B9). These seedlings were grown in pesticide-free vineyards maintained by the New York State Agricultural Experiment Station, Geneva, New York, USA.

### Predator abundance characterization

The abundance of *T*. *pyri*, was measured as mean counts per leaf as follows: five mid-shoot leaves from a single field-grown vine were sampled on each occasion. Phytoseiid motiles (adults and hatched immature mites) were counted by examining each leaf under a microscope. To confirm identification, up to 10 adult phytoseiids per plant from each sampling occasion were mounted on glass slides and observed under the microscope. Each genotype was sampled five times in 2005 (twice in June, once in July, August, and September), five times in 2006 (twice in June, once on July, August, and September), twice in 2007 (June and August), and three times in 2008 (June, July, and August). For each year, phenotypes were determined as the average value of mean counts per leaf.

### Leaf trichome characterization

Leaf trichomes were characterized in parents and the F_1_ progeny using six variables: (1) domatia size (mm), (2) domatia rating, based on the density of trichomes that compose each individual domatia as described in refs. ^30,44^, (3) density of bristles on the leaf blade, (4) density of bristles along leaf veins, (5) density of hairs on the leaf blade, and (6) density of hairs along leaf veins. Trichomes were observed under a microscope and classified as either bristles (trichomes shorter than 0.25 mm and upright) or hairs (trichomes longer than 0.25 mm and prostrate), and rated based on a scale from 0 (absent) to 9 (very dense)^45^. The bristle ranking corresponds to the number of bristles. Rank 1 was assigned to the presence of 0–4 bristles, rank 3 between 5 and 24 bristles, rank 5 between 25 and 42 bristles, rank 7 between 43 and 60 bristles, and a rank of 9 above 61 bristles. The hair ranking corresponds to the number of times a hair crossed over the top of the vein on four 1 cm square of adaxial leaf surface. Rank 1 was assigned to the presence of 0–1 hairs, rank 3 between 2 and 4 hairs, rank 5 between 5 and 8 hairs, rank 7 between 9 and 13 hairs, and a rank of 9 above 13 hairs. The bristle scoring system was applied to four vein axils per leaf to generate a mean leaf domatia rating. Domatia size was determined by measuring the diameter of the zone of bristles extending away from the same four domatia with a micrometer and calculating a mean size per leaf. Trichome parameters were assessed by collecting 2–4 mid-shoot leaves per plant once during 2004, 2005, and 2008 (190, 155, and 152 plants, respectively). Parental trichome parameters were measured as described above, using ten leaves per parent collected in 2005.

### Single nucleotide polymorphism (SNP) genotyping and construction of genetic maps

Dense genetic maps were previously constructed and described^46^. Briefly, one young leaf per vine was used for DNA extraction using the DNeasy® 96 Plant Kit (Qiagen) amended with 2.8% w/v PVP-40 in the extraction buffer. Samples were used for 384-plex library construction at the Cornell Institute for Genomic Diversity following a genotyping-by-sequencing protocol^47^. Libraries were sequenced with an Illumina HiSeq 2000 DNA sequencer (single-end, 100 bp read length) at the Genomics Facility of the Institute of Biotechnology at Cornell University.

Raw sequence data were aligned to the grapevine PN40024 reference genome version 12X.0^48,49^ and processed into SNP genotype files in VCF format using the TASSEL 3.0 GBS pipeline^50^. SNP names indicate their position on the reference genome, coded as S(chromosome)_(position in bp). Vines resulting from self-pollination or cross contamination were identified using relatedness and paternal/maternal Mendelian incompatibility ratios. SNPs from the remaining samples were filtered down to a subset of pseudo-testcross markers^51^, according to quality score, read depth and minor allele frequency (MAF). Parental genetic maps were constructed using a de novo pipeline^46^. Parental maps were compared based on the physical position of SNP markers in the grapevine reference genome PN40024, as the use of exclusively pseudo-testcross markers does not allow the construction of consensus maps.

### QTL analysis

QTL were determined using the R/qtl package^52^ implemented in the statistical software R^53^. Phytoseiid abundance data were power transformed as described in the Statistics section below. Multipoint probabilities were calculated using *calc*.*genoprob* with step = 1 and default parameters. Initial QTL positions were determined with the *scanone* function using a normal model, Haley-Knott regression and default parameters. LOD significance scores were determined by permutation tests (1000). Initial QTL positions were then used to define QTL with the *makeqtl* function, the significance of model terms was tested with the *fitqtl* command, and positions were refined with *refineqtl*. Models were constructed by adding and then removing QTL one at the time, and the model with the best fit was selected. Non-significant QTL or non-significant QTL interactions were also removed. The *addqtl* command was used to test if another QTL was needed, which was then added to the model as described above. A histogram of the model’s residuals was used to check for normality. A 1.5 LOD support interval, defined as the interval in which the LOD score is within 1.5 units of its maximum^54^, was determined using *lodint* function and QTL effects were calculated as the difference in the mean phenotype value of individuals within each genotype class at the marker or pseudomarker with the highest LOD score, using *effectplot*. QTL names follow R/qtl nomenclature: (linkage group)@(position in cM). QTL located at similar physical positions on both genetic parental maps are reported as co-localized but separate QTL, because the use of exclusively pseudo-testcross markers does not allow the construction of consensus maps.

### Statistics

Statistical analyses were performed using the statistical software R^53^. Parental phenotypes were compared using a *t*-test. Correlations among progeny phenotype data collected as described above (Pearson’s r) were determined using the *cor.test* function from the stats package and visualized using the corrplot package. Normality of trait distribution was tested using the *shapiro.test* function from stats package. The lambda parameters for Box-Cox power transformation were determined using the *boxcoxnc* function from the AID package in R^55^.

### Candidate genes

A total of 28 genes involved in non-glandular trichome development were obtained from the TrichOME database and other sources^32,33,56^ (Additional file 1). Protein–protein BLAST^57^ was performed between candidate gene products (Additional file 2) and both the V1 and V2.1 annotation of the 12x.0 version of the PN40024 reference genome^37^. Hits within QTL supported intervals were selected with the GenomicRanges package in R^58^. *Vitis* candidate gene products were aligned to RefSeq protein - *Arabidopsis thaliana* in the NCBI database using BLASTp to confirm similarity of function.

## Supplementary information

Arabidopsis thaliana genes

BLASTp statistics between Arabidopsis thaliana gene products
