# Supplementary material for: A QTL associated with leaf trichome traits has a major influence on the abundance of the predatory mite Typhlodromus pyri in a hybrid grapevine population
Source: Hortic Res. 2019 Jul 21;6:87. doi: 10.1038/s41438-019-0169-8 (PMC6804712; doi:10.1038/s41438-019-0169-8)
Supplement: Supplementary file 1 — Arabidopsis thaliana genes [file 41438_2019_169_MOESM1_ESM.pdf]

Additional file 1 - *Arabidopsis thaliana* genes involved in the trichome developmental pathway

| Gene Name | Description | NCBI Protein accession number |
|---|---|---|
| ATMYBL2 | MYB-LIKE 2; DNA binding / transcription factor | [NCBI:NP_177259.1] |
| ATMYC1 | MYC-RELATED TRANSCRIPTION FACTOR 1; DNA binding / transcription factor | [NCBI:NP_001154194.1] |
| CPC | CAPRICE; DNA binding / transcription factor | [GenBank:AAS09991.1] |
| CPR5 | CONSTITUTIVE EXPRESSION OF PR GENES 5 | [NCBI:NP_569003.1] |
| EGL3 | ENHANCER OF GLABRA3; DNA binding | [NCBI:NP_001185302.1] |
| ETC1 | ENHANCER OF TRY AND CPC 1; DNA binding / transcription factor | [NCBI:NP_171645.1] |
| ETC2 | ENHANCER OF TRY AND CPC 2 | [NCBI:NP_850145.1] |
| ETC3 | ENHANCER OF TRY AND CPC 3; DNA binding / transcription factor | [NCBI:NP_974493.1] |
| GAI | GA INSENSITIVE; transcription factor | [NCBI:NP_172945.1] |
| GIS | GLABROUS INFLORESCENCE STEMS; nucleic acid binding / transcription factor/ zinc ion binding | [NCBI:NP_191366.1] |
| GIS2 | GLABROUS INFLORESCENCE STEMS 2; nucleic acid binding / transcription factor/ zinc ion binding | [NCBI:NP_196283.1] |
| GL1 | GLABRA 1; transcription factor | [NCBI:NP_189430.1] |
| GL2 | GLABRA 2; DNA binding / transcription factor | [NCBI:NP_001185443.1] |
| GL3 | GLABRA 3; transcription factor | [NCBI:NP_680372.1] |
| MYB23 | MYB DOMAIN PROTEIN 2; DNA binding / transcription factor | [NCBI:NP_198849.1] |
| MYB5 | TRANSCRIPTION REPRESSOR MYB5 | [NCBI:NP_187963.1] |
| RGA1 | REPRESSOR OF GA1-3 1; transcription factor | [NCBI:NP_178266.1] |
| RGL1 | RGA-LIKE 1; transcription factor | [NCBI:NP_176809.1] |
| SAD2 | SUPER SENSITIVE TO ABA AND DROUGHT 2; protein transporter | [NCBI:NP_180724.2] |
| SPL8 | SQUAMOSA PROMOTER BINDING PROTEIN-LIKE 8; DNA binding | [NCBI:NP_683267.1] |
| SPY | SPINDLY; transferase, transferring glycosyl groups | [NCBI:NP_187761.1] |
| TCL1 | TRICHOMELESS1; DNA binding | [NCBI:NP_001031445.1] |
| TCL2 | TRICHOMELESS 2 | [NCBI:NP_001118417.1] |
| TRY | TRIPTYCHON; DNA binding / transcription factor | [NCBI:NP_200132.2] |
| TT8 | TRANSPARENT TESTA 8; DNA binding / transcription factor | [NCBI:NP_192720.2] |
| TTG1 | TRANSPARENT TESTA GLABRA 1; nucleotide binding | [NCBI:NP_851069.1] |
| TTG2 | TRANSPARENT TESTA GLABRA 2; transcription factor | [NCBI:NP_181263.2] |
| ZFP8 | ZINC FINGER PROTEIN 8; nucleic acid binding / transcription factor/ zinc ion binding | [NCBI:NP_181725.1] |
