# Supplementary material for: A QTL associated with leaf trichome traits has a major influence on the abundance of the predatory mite Typhlodromus pyri in a hybrid grapevine population
Source: Hortic Res. 2019 Jul 21;6:87. doi: 10.1038/s41438-019-0169-8 (PMC6804712; doi:10.1038/s41438-019-0169-8)
Supplement: Supplementary file 2 — BLASTp statistics between Arabidopsis thaliana gene products [file 41438_2019_169_MOESM2_ESM.pdf]

Additional file 2 - BLASTp statistics between *Arabidopsis thaliana* gene products involved in the trichome regulatory pathway (Additional file 1) on to predicted proteins of *Vitis vinifera* PN40024 genome (Version 2.1, Vitulo et al., 2014) located within phytoseiid and trichome abundance quantitative trait loci (QTL) supported intervals.

**Candidate Gene (*Vitis*)**	**Query (*A thaliana* in trichome pathway)**	**Query gene name**	**Identity**	**Alignment length**	**# Mismatch**	**Gap opens**	**e-value**	**Bit score**	**Associated QTL**
VIT_201s0127g00730.1	GI 15232860 ref NP_189430.1	GL1	76.36	110	26	0	$8.0*10^{-55}$	178	Phytoseiid Hairs on blade
VIT_201s0127g00730.1	GI 15242649 ref NP_198849.1	MYB23	77.27	110	25	0	$4.0*10^{-58}$	186	Phytoseiid, Hairs on blade
VIT_201s0026g00850.1	GI 15240118 ref NP_196283.1	GIS2	36.27	102	46	1	$2.0*10^{-14}$	70	Phytoseiid, Domatia (rating), Domatia (size), Hairs on blade, Hairs on veins
VIT_201s0026g00850.1	GI 15227472 ref NP_181725.1	ZFP8	35.04	117	63	3	$9.0*10^{-12}$	64	Phytoseiid, Domatia (rating), Domatia (size), Hairs on blade, Hairs on veins
VIT_201s0010g02270.1	GI 15226311 ref NP_178266.1	RGA1	34.09	396	227	11	$4.0*10^{-69}$	236	Phytoseiid, Domatia (rating), Bristles on blades, Bristles on veins.
VIT_201s0010g02270.1	GI 15219630 ref NP_176809.1	RGL1	34.79	388	229	10	$9.0*10^{-73}$	244	Phytoseiid, Domatia (rating), Bristles on veins, Bristles on blades
VIT_02s0012g02030.t01	GI 334184032 Ref NP_001185443.1	GL2	38.95	742	374	20	$7.0*10^{-161}$	490	Hairs on veins
VIT_05s0077g01390.t01	GI 15240118 ref NP_196283.1	GIS2	40.54	74	34	1	$1.0*10^{-11}$	60	Hairs on blades
VIT_208s0007g06870.1	GI 15230939 ref NP_191366.1	GIS	44.28	271	100	14	$2.0*10^{-48}$	163	Hairs on blades
VIT_208s0007g06870.1	GI 15240118 ref NP_196283.1	GIS2	41.2	233	92	9	$2.0*10^{-37}$	132	Hairs on blades
VIT_208s0007g06870.1	GI 15227472 ref NP_181725.1	ZFP8	42.91	275	102	11	$3.0*10^{-50}$	168	Hairs on blades

**Candidate Gene (*Vitis*)**	**Query (*A thaliana* in trichome pathway)**	**Query gene name**	**Identity**	**Alignment length**	**# Mismatch**	**Gap opens**	**e-value**	**Bit score**	**Associated QTL**
VIT_208s0007g07230.1	GI 15231271 ref NP_187963.1	MYB5	56.08	255	67	7	$2.0*10^{-85}$	261	Hairs on blades
VIT_15s0021g02290.t01	GI 22329284 ref NP_683267.1	SPL8	52.8	125	45	2	$3.0*10^{-33}$	128	Domatia (size)
VIT_215s0021g02300.1	GI 22329284 ref NP_683267.1	SPL8	50.42	355	103	12	$2.0*10^{-88}$	272	Domatia (size)
VIT_15s0048g02000.t01	GI 334184032 ref NP_001185443.1	GL2	41.76	795	371	17	0	588	Domatia (size), Domatia (rating), Bristles on blades
VIT_215s0046g00170.1	GI 15231271 ref NP_187963.1	MYB5	62.35	162	45	1	$8.0*10^{-66}$	209	Domatia (rating)
VIT_215s0046g01130.1	GI 41618962 gb AAS09991.1	CPC	62.35	85	31	1	$2.0*10^{-27}$	99	Domatia (rating)
VIT_215s0046g01130.1	GI 30684581 ref NP_850145.1	ETC2	65.15	66	23	0	$5.0*10^{-25}$	93	Domatia (rating)
VIT_215s0046g01130.1	GI 42572793 ref NP_974493.1	ETC3	63.89	72	25	1	$2.0*10^{-23}$	88	Domatia (rating)
VIT_215s0046g01130.1	GI 79323486 ref NP_001031445.1	TCL1	70.91	55	16	0	$3.0*10^{-23}$	88	Domatia (rating)
VIT_215s0046g01130.1	GI 186504271 ref NP_001118417.1	TCL2	55.42	83	36	1	$2.0*10^{-23}$	89	Domatia (rating)
VIT_215s0046g01130.1	GI 30696297 ref: NP_200132.2	TRY	70.73	82	24	0	$2.0*10^{-31}$	110	Domatia (rating)
